# A Generalized Deforestation and Land-Use Change Scenario Generator for Use in Climate Modelling Studies

**DOI:** 10.1371/journal.pone.0136154

**Published:** 2015-09-22

**Authors:** Adrian Mark Tompkins, Luca Caporaso, Riccardo Biondi, Jean Pierre Bell

**Affiliations:** 1 Earth System Physics, The Abdus Salam International Centre for Theoretical Physics (ICTP), Strada Costiera 11, Trieste, Italy; 2 Wegener Center for Climate and Global Change, University of Graz, Brandhofgasse 5, Graz, Austria; 3 Centre of Atomic Molecular Physics and Quantum Optics University of Douala, P.O. Box.8580, Douala, Cameroon; The Ohio State University, UNITED STATES

## Abstract

A new deforestation and land-use change scenario generator model (FOREST-SAGE) is presented that is designed to interface directly with dynamic vegetation models used in latest generation earth system models. The model requires a regional-scale scenario for aggregate land-use change that may be time-dependent, provided by observational studies or by regional land-use change/economic models for future projections. These land-use categories of the observations/economic model are first translated into equivalent plant function types used by the particular vegetation model, and then FOREST-SAGE disaggregates the regional-scale scenario to the local grid-scale of the earth system model using a set of risk-rules based on factors such as proximity to transport networks, distance weighted population density, forest fragmentation and presence of protected areas and logging concessions. These rules presently focus on the conversion of forest to agriculture and pasture use, but could be generalized to other land use change conversions. After introducing the model, an evaluation of its performance is shown for the land-cover changes that have occurred in the Central African Basin from 2001–2010 using retrievals from MODerate Resolution Imaging Spectroradiometer Vegetation Continuous Field data. The model is able to broadly reproduce the spatial patterns of forest cover change observed by MODIS, and the use of the local-scale risk factors enables FOREST-SAGE to improve land use change patterns considerably relative to benchmark scenarios used in the latest Coupled Model Intercomparison Project integrations. The uncertainty to the various risk factors is investigated using an ensemble of investigations, and it is shown that the model is sensitive to the population density, forest fragmentation and reforestation factors specified.

## 1 Introduction

Deforestation has long been considered a critical issue for the future preservation of ecosystems and reducing CO_2_ emissions. Additionally, many studies highlight the strong impact that land-use change (LUC) can have on both the local and regional climate through albedo and surface flux changes as well as the indirect CO_2_ response, using both regional and global models [1–5]. Understanding the effects of land-use and land-cover change (LULCC) on climate is crucial if human influence on climate and the potential effectiveness of land-use (LU) based mitigation strategies such as reforestation or biofuels are to be assessed.

Deforestation estimates are uncertain and vary considerably despite inexorably improving remote sensing technology. Taking Central Africa as an example, estimates of deforestation rates are 0.53% per year in southern Cameroon [6], while Duveiller et al. [7] used Landsat images to estimate 0.21% per year for Central Africa, rates that are two and four times lower than for South America and Asia, respectively. In contrast, Hansen et al. [8] applied the MODerate Resolution Imaging Spectroradiometer (MODIS) for deriving forest cover products to calibrate Landsat data in the Congo River Basin and found a deforestation rate lower than 0.1% per year. Zhang et al. [9] analyzed Landsat TM images conclude that from the 1980s to 1990s the annual rate of deforestation was 0.42% varying from 0.03% to 2.72% in the Congo Basin, which is similar to the previously reported values of 0.41% [10]. A recent FAO report on the rainforest [11] gives the annual deforestation rate for the period 2000–2010 as 0.23% in the Congo Basin, about 0.43% in the Amazon Basin and about 0.41% in the Southeast Asia. Nevertheless, despite these varying estimates the consensus is that tropical forest systems are at risk [12].

Estimating factors that drive LUC is also challenging, since these can vary from region to region, and the factors are multiple and interacting [13–15]. Local scale drivers of deforestation are often related to access to local markets (distance to the nearest roads and population centres) and the population density that drives demand [16–18]. Soil quality and terrain slope will determine land productivity. Non local markets can also drive LUC [14, 15] if access to ports is adequate and climate is suitable for high value, export-oriented crops. Related to this, if LUC in the form of deforestation is driven by wood harvesting demands [19, 20], the value of the tree species themselves can have an obvious impact.

Existing forest degradation and fragmentation increase access and thus deforestation rates [14]. In terms of national legislation, granting of logging concessions or conversely establishment of national parks or other protective measures are very important [21–27].

To complicate matters further, macro-scale factors are also at play with global economic conditions and regional legislation and policy driving external deforestation demand, with land cleared for livestock rearing in Brazil for example [28–30], or EU policy on biofuel subsidies driving clearance for oil palm plantations in Asia [31, 32]. National or international policy for protection (e.g. the United Nations collaborative initiative on Reducing Emissions from Deforestation and forest Degradation UN-REDD) or Central Africa Regional Program for the Environment (CARPE) in the Congo Basin can also reduce deforestation rate, (e.g. halved in the Central Africa in designated reserve areas [21, 22, 26, 33]).

In order to understand how this complex web of drivers affects present day deforestation and possibly predict future changes in LU it is useful to derive models. Many deforestation models are derived locally using regressions between observed deforestation rates and the local predictor values [6, 34, 35]. Thus the influence of local factors determine the spatial pattern of deforestation, while the unspecified global factors are implicitly included in the overall deforestation rates, but are necessarily time invariant. Such models can be accurate on a national or regional scale and are useful to define risk maps for short-term deforestation rates. However, they are unlikely to be able to provide future scenarios of deforestation as the local and global conditions may change considerably. Moreover, the great variability in driving determinants between locations means that such models cannot be applied to other regions without re-deriving the regressions coefficients, and it is difficult to incorporate different scenarios for future macroscale deforestation drivers.

The lack of generality of the deforestation models has inhibited the development of generalized deforestation scenario generators for use in climate studies. Another concern is the apparent mismatch of scales with many of the deforestation models working on very fine spatial scales, with the impact of roads and towns determined to have an e-folding spatial impact scale of 𝒪(10 km) [36, 37] which implies that factors are essentially subgrid-scale relative to current generation of global climate models. This has meant that until recently, studies of interactions of deforestation and climate were often idealized, where large-scale deforestation is applied as a discrete step change and the model run to equilibrium to gauge the impact on local climate [5, 38, 39]. While useful for gauging LUC-driven climate sensitivity, such idealized experiments preclude the investigation of gradual realistic forest change, which could reveal the existence of climate “tipping points” where local climates could switch discretely from one state to another at a certain critical deforestation or LUC [40, 41]. Such behavior is not considered unlikely as the relationship between climate and LU could be highly-nonlinear, as illustrated in the seminal paper of Charney [42].

More recently, a number of projected scenarios for future LU have been developed by various modelling groups [43–45]. The impacts assessment modelling framework of the fifth assessment report of the Intergovernmental Panel on Climate Change (IPCC) produced four sets of LU maps associated with each of the four greenhouse gas representative concentration pathways (RCPs) [46–49]. These formed the basis of an optional experimental line for the climate modelling groups to examine the impact of realistic LUC. Twin climate model runs were conducted with and without anthropogenically driven LUC to assess its impact on climate relative to greenhouse gas emissions. In the climate model experiments, anthropogenic LUC had a limited impact on climate [50, 51].

**Fig 1:**
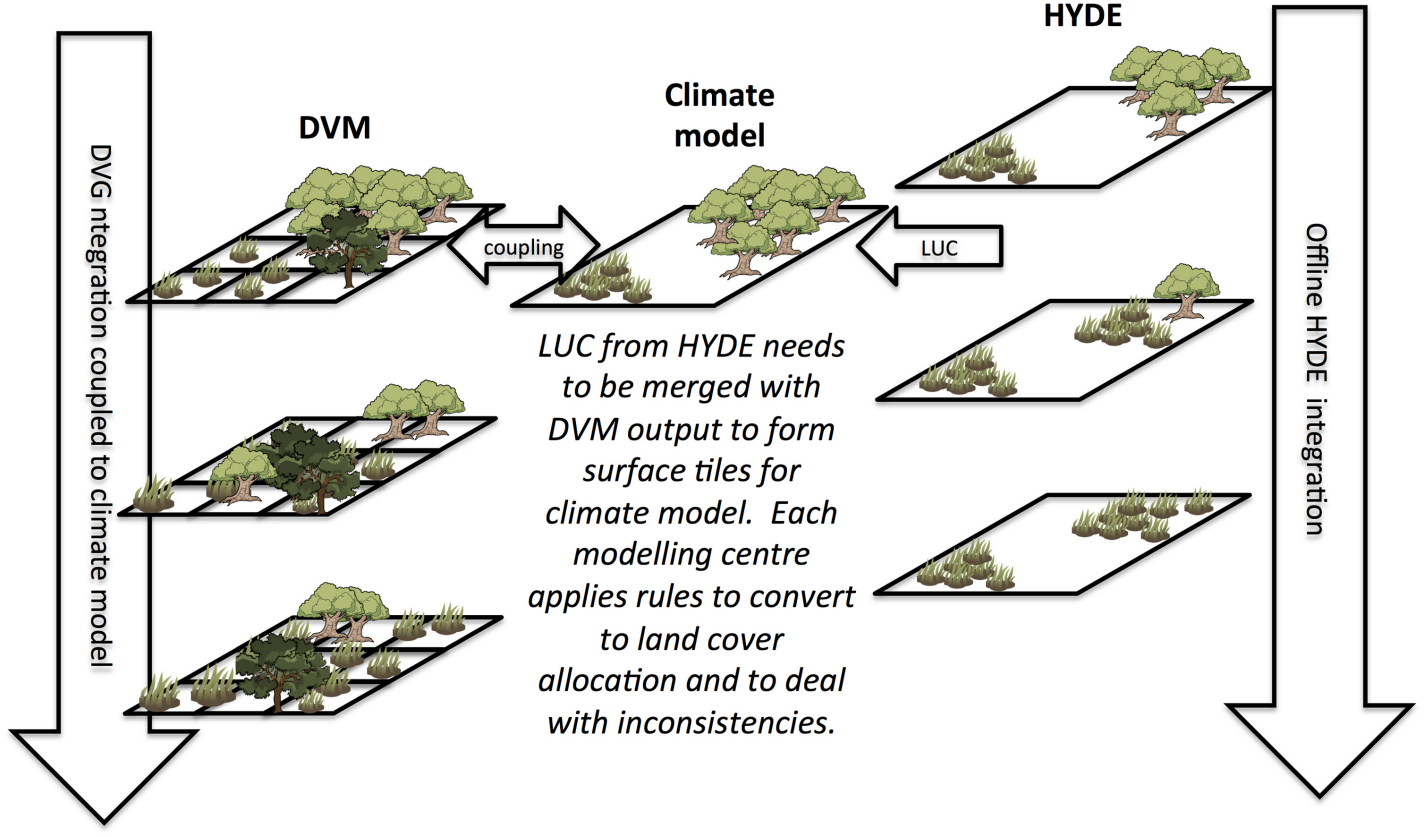
Schematic of HYDE integration into a coupled climate-land surface model. The method employed to convert the five HYDE categories into land categories used by ESMs is not straight-forward and a mismatch between the land surface model and the HYDE 3.1 land cover classification can appear.

In order to set up these LUC experiments, a number of technical challenges had to be addressed. Firstly, the integrated assessment models (IAMs) provided very different information in terms of LUC (with one model only providing national averages for example). Thus the HYDE model was used to consistently convert the IAM LU information to a 2×2 degree grid of 5 basic LUC types, which were further disaggregated to 0.5 degree resolution [52]. However, the HYDE model output still required interpolation to the climate model grid with the land transitions translated to each climate model’s respective land cover classes. Moreover, as illustrated schematically in Fig 1, the offline nature of the HYDE LU transitions complicates the task of simultaneously employing an online dynamical global vegetation model (DGVM), since various inconsistencies may arise. For example, the HYDE model may allocate deforestation to satisfy wood harvesting demands in a cell in which the DGVM has already simulated forest die-back due to climate change. In such cases, models must implement ad hoc rules to spatially reallocate land use trends, which may vary from model to model.

In order to increase the consistency of the modelling approach between climate modelling groups, account for local scale drivers of LUC, and most importantly permit fully coupled climate model integrations that account for anthropogenic LUC while using a dynamic vegetation model, this work presents a new model for disaggregating coarse scale or national level land use change information directly on the fine grid resolution of widely used digital vegetation models.

The FOREST-SAGE model will attempt to include local determinants of deforestation variables such as access to market, population density, transport network and forest fragmentation [14] in a reasonable but idealized way compatible with previous models to provide spatial distributions of deforestation. However, rather than applying regression coefficients for the overall rate of deforestation, the gross regional deforestation rates will be externally specified, for example by idealized function of the time or by an ensemble of economically and politically driven land use change scenarios (e.g. [52]), in a similar vein to the IPCC AR5 future emissions scenarios.

**Fig 2:**
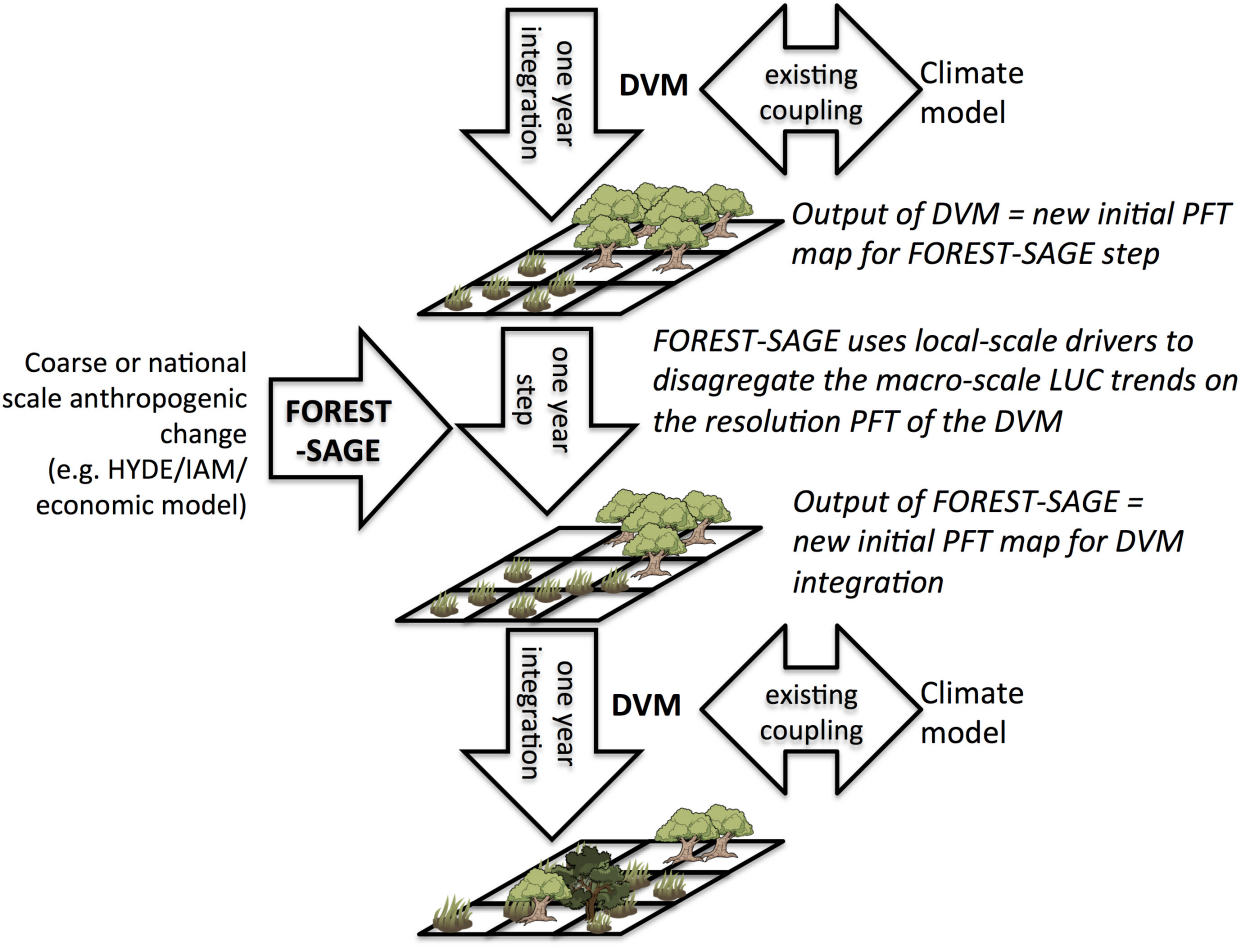
Schematic of FOREST-SAGE integration into a coupled climate-land surface model. The schematic emphasizes how FOREST-SAGE translates global anthropogenic land-use scenarios to ESMs grid-scale land cover on-line in a fully coupled way.

The model provides output directly in terms of the plant function types employed by the LU scheme coupled to the climate model. By doing this, the model can be integrated on-line, in particular this allows the integration simulations with a DGVM, as shown in Fig 2, where the climate-DGVM coupled run calls FOREST-SAGE once per year, passing the DGVMs PFT map as initial conditions. FOREST-SAGE then allocates the coarse scale anthropogenic land use change information (for example, provided by HYDE at 2×2 degree resolution) to the fine-scale DGVM resolution using local drivers. The resulting modified map of PFTs percentages in each cell is passed back to the DVGM to initialize the next one year coupled integration. In this way, the coupling between the climate model and DGVM is unmodified, and the anthropogenic LUC is incorporated consistently. In this paper, the model is presented and then evaluated by simulating present day LUC in the Congo.

## 2 Materials and Methods

### 2.1 FOREST-SAGE model description

The FOREST-SAGE model is designed to be flexible and can run on a variety of resolutions and can be initialized using satellite observations or dynamical vegetation models PFT maps. The version used in this study is available in S1 File. The model can be operated regionally or even locally with higher resolution input. The focus here is on deforestation but the technique could be applied to a spectrum of LUC types. For each region of interest Ω, annual macro-region deforestation rates (*M*_Ω_) are defined according to a future scenario such as “business as usual”. The factor *M*_Ω_ allows the model to differentiate between greatly differing economic and policy drivers of deforestation between regions, which result in the higher rates in Asia compared to, say, Africa. The deforestation rate is specified as a function of time *M*_Ω_(*t*) in each deforestation scenario, which may account for potential future policy changes or economic/population developments. In fact, socio-economic factors associated with deforestation remain poorly understood [14], partly because different factors operate unevenly in different countries and partly because of the scarcity of reliable data [15, 53].

In the illustrative example in this initial work, the deforestation rate will be static and equal to the observational derived deforestation rate. However, the deforestation rate could alternatively be dynamically specified as a function of global or regional forecast cover, reflecting the potential introduction of more stringent conservation legislation in response to future lower forest cover e.g. large-scale die-back simulated as part of an Integrated Assessment modelling framework [54].

In summary, rather than simply extending past LUC rates into the future as regression models, FOREST-SAGE has the potential to generate ensembles of LUC scenarios to evaluate risk assessment ranging from business as usual to drastically reduced deforestation to investigate the impact of a wide variety of potential pathways. The integration time step for FOREST-SAGE is one year. The model predicts an annual deforestation risk factor for each and every forested grid-cell in the model domain (with the focus here on tropical regions), according to the *local* risk factors outlined below. The deforestation rate is proportional to risk in each cell, but scaled to give the macro rate *M*_Ω_(*t*) for each area. If the model is operated offline, this new modified map after one year is used to initialize the following year’s time-step and FOREST-SAGE thus generates a series of annual potential land cover states. However, FOREST-SAGE can be fully coupled with a climate model using a dynamic vegetation model in which case land cover classes would also be updated by these models.

**Fig 3:**
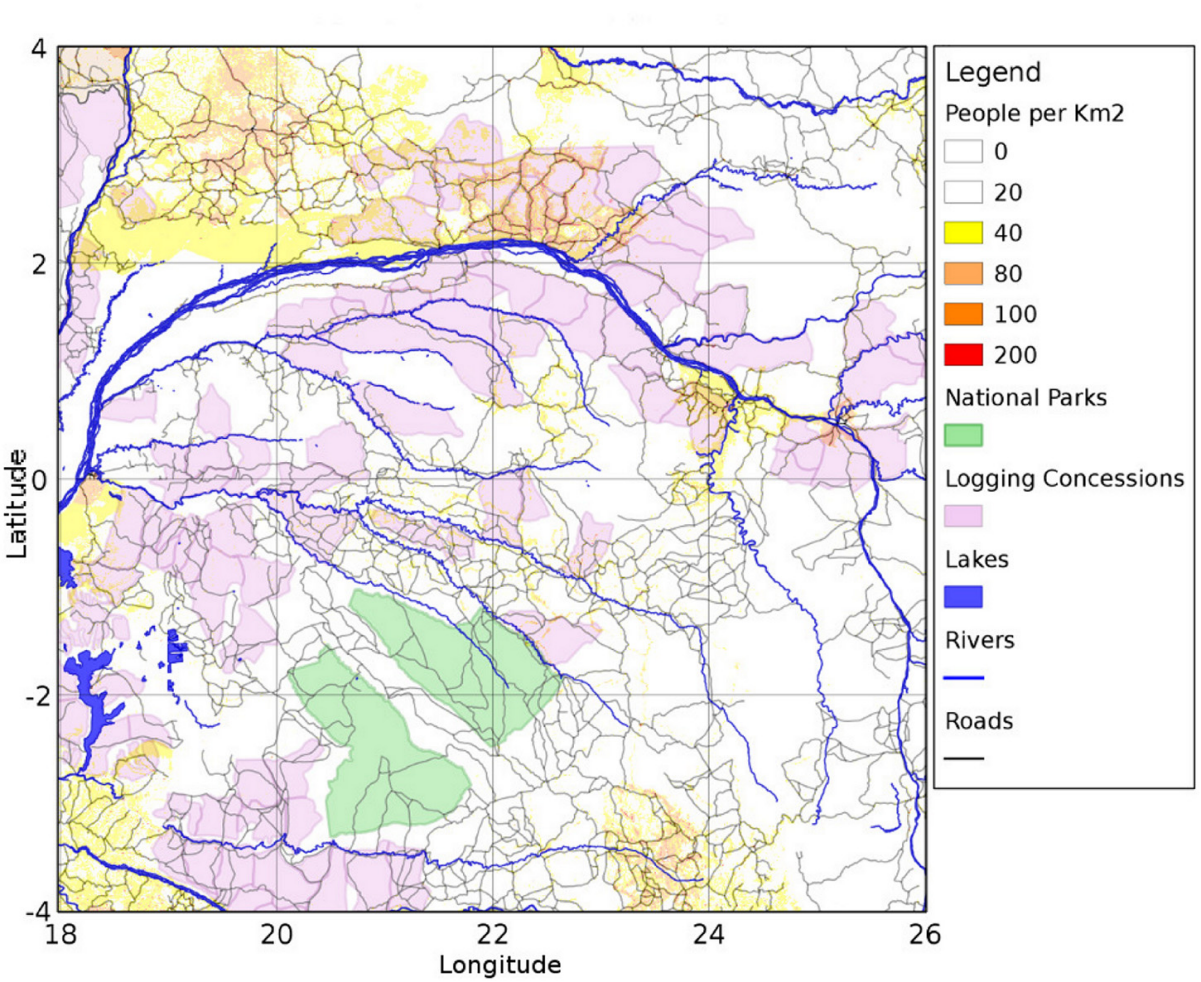
Overview of the local drivers’ spatial distribution. The input data used to initialize the model are summarized in Table 1.

In FOREST-SAGE there are several factors (Fig 3) that affect the local distribution of deforestation risk (*r* ^*tot*^), namely the proximity to roads (*r* ^*road*^) and rivers (*r* ^*river*^) that permit access to markets, proximity to population centres and their population density (*r* ^*pop*^), location within protected areas designated under national or international agreements (*r* ^*park*^) or within designated logging concessions (*r* ^*log*^), which determines access, and lastly the existing local forest cover fraction itself, (*r* ^*frag*^). Presently, factors that influence land suitability for agriculture such as soil type or terrain slope that can play an important role in the deforestation/regrowing [55, 56] are not accounted for but may be easily incorporated into the flexible framework. The deforestation risk, at each grid-cell location *i*, associated with each of these factors are multiplicatively combined to provide a total deforestation risk:

$$\begin{array}{c} r_{i}^{tot}=r_{i}^{road}r_{i}^{pop}r_{i}^{river}r_{i}^{park}r_{i}^{log}r_{i}^{frag}. \end{array}$$ (1)

**Fig 4:**
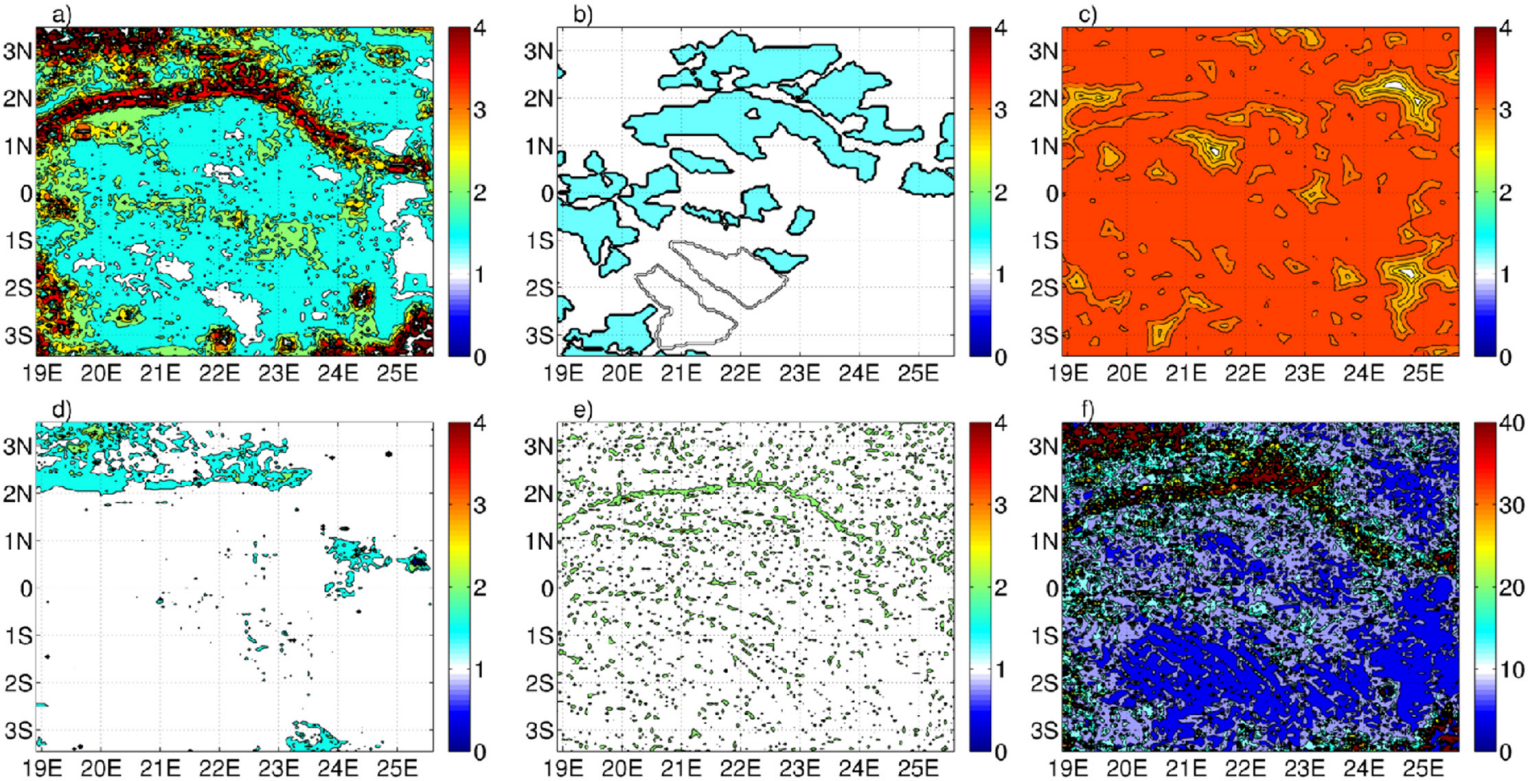
Deforestation risk associated to the experiment 1 (Table 3). The panel *a* is referred to the forest cover risk (*r* ^*frag*^), while the panel *b* to the risk related both to the protected area (*r* ^*park*^) and to the logging concessions (*r* ^*log*^). In the panel *c* is shown the risk associated to the roads (*r* ^*road*^) while the panel *d* is connected to the population centres risk (*r* ^*pop*^). Finally the panel *e* is related to the river risk (*r* ^*river*^) and lastly in the panel *f* is shown the global deforestation risk (*r* ^*tot*^).

The individual factors are not normalized since they are combined multiplicatively, thus it is the relative change of risk across the determining input factors that is relevant (Fig 4). A simple example is the risk associated with national parks, where the relevant parameter is the *ratio* of risk between being located within or outside of a protected area.

Once $r_{i}^{tot}$ is determined for each location *i* of the input plant function type (PFT) map, the forest cover change is calculated as:

$$\begin{array}{c} \frac{\partial f}{\partial t}\equiv M_{\Omega }\sum_{i=1}^{N_{\Omega }}f_{i}=\sum_{i=1}^{N_{\Omega }}D_{i}+\sum_{i=1}^{N_{\Omega }}A_{i}, \end{array}$$ (2)

where *D*_*i*_ is the gross deforestation rate proportional to the risk and equal to:

$$\begin{array}{c} \sum_{i=1}^{N_{\Omega }}D_{i}=\alpha_{\Omega }\sum_{i=1}^{N_{\Omega }}r_{i}^{tot}f_{i}. \end{array}$$ (3)

In Eq 3, *α*_Ω_ is the scale factor derived for each macro-zone Ω:

$$\begin{array}{c} \alpha_{\Omega }\left(t\right)=\frac{M_{\Omega }\left(\sum_{i=1}^{N_{\Omega }}f_{i}\right)-\sum_{i=1}^{N_{\Omega }}A_{i}}{\sum_{i=1}^{N_{\Omega }}r_{i}^{tot}f_{i}}, \end{array}$$ (4)

where *A*_*i*_ is the reforestation rate, the index *i* is a function of macro-region Ω to emphasize that the summation is performed only for the points *N*_Ω_ lying with each macro deforestation zone and *f*_*i*_ is the percent forest cover in location *i*. For brevity hereinafter Ω will be implied in the equations.

For each of the drivers the simplest mathematical function possible has been chosen in order to minimize the degrees of freedom. Each driver is then governed by two variables that describe the relative magnitude of the maximum impact (*k*) that the drivers can have on LUC and their spatial influence (*l*). The local deforestation impacts are now introduced in turn.

#### Forest cover

The deforestation rate is a function of the forest cover, *f*, itself. If the forest cover is zero then obviously no deforestation can take place. Likewise previous work has shown that forest fragmentation can lead to increased human access and consequentially much greater deforestation along the forest boundaries [20, 21]. The edges can extend deep into remaining forest areas increasing the number of forest fragments that can lead, for example, an increase of the edge-related fires that can penetrate up to few kilometers into fragmented forest [20].

Using an edge feedback model, Cumming et al. [57] showed that edges can significantly amplify the effects of deforestation (with a maximum edge effect when the forest cover is 50%) leading to a rapid deforestation rate (up to 3-4 times faster than those occur under a linear deforestation model). Thus, in our implementation, the deforestation risk increases from zero with no forest cover to a maximum at intermediate cover, then reducing to zero with 100% forest cover. This is incorporated into the model by first calculating a distance weighted forest density for each grid-cell:

$$\begin{array}{c} {\bar{f}}_{i}=\frac{\sum_{j=1}^{N_{\Omega }}f_{j}e^{-d_{i,j}/l_{f}}}{\sum_{j=1}^{N_{\Omega }}e^{-d_{i,j}/l_{f}}}, \end{array}$$ (5)

where *d*_*i*,*j*_ is the distance between locations *i* and *j* and *l*_*f*_ is the distance weighting, set to 2.5 km. Next, a function based on a symmetrical beta distribution is applied:

$$\begin{array}{c} r_{i}^{frag}=\left(k^{frag}-1\right)\frac{{f_{i}}^{p}{\left(1-\bar{f_{i}}\right)}^{p}}{0.5^{2p}}+1, \end{array}$$ (6)

where *p* is the beta function shape parameter which determines how quickly deforestation risk changes with increasing fragmentation. In the above expression $\bar{f_{i}}$ has been used in the second term on the RHS to increase risk near forest edges, while *f* is used in the first term to relate forest risk directly to the local cover when sparse coverage remains (edge effects are less important). The maximum risk occurs when mean homogeneous forest cover is 50%, hence the normalization factor which ensures that the risk is equal to *k* ^*frag*^ at this value.

#### Roads and Rivers

Roads increase land value by permitting access to markets and can drastically increase deforestation rates. A classic example of this effect was the 1960s construction of the coastal road in north east Brazil which greatly accelerated the deforestation rates in the coastal region [58]. The impact of the road on deforestation falls as a function of the distance to the road and thus is parameterized as:

$$\begin{array}{c} r_{i}^{road}=(k^{road}-1)e^{-d_{i}^{road}/l_{road}}+1 \end{array}$$ (7)

where $d_{i}^{road}$ is the distance of location *i* to the nearest road, and *l*_*road*_ is the exponential decay of the impact of roads. For simplicity, no distinction is made between minor or major roads or between paved or unpaved roads: it is only the distance and thus ease of access to the road network, per se, that is important. Setting the value for *l*_*road*_ is not straightforward as regression models indicate a wide range of values depending on the region in question and indicates that roads have a small scale impact, with the majority of deforestation occurring in narrow corridors of 𝒪 (10 km) along newly built roads [59]. However roads can influence deforestation rate at distance of many tens of kilometers [60]. Southworth et al. [59] using multi-temporal Landsat images evaluated the deforestation impact of the new Inter-Oceanic Highway over the border between Peru, Brazil and Bolivia. The results of this analysis showed values of *l*_*road*_ ranging from few kilometers up to around 45 km depending on the development of the area. FOREST-SAGE presently uses the current road network in its calculations and does not yet contain a parameterization to add new roads with population development.

Similarly to the roads, rivers can provide an easier access to the forest, [61, 62] and the deforestation “risk” associated with them is parametrized as:

$$\begin{array}{c} r_{i}^{river}=(k^{river}-1)e^{-d_{i}^{river}/l_{river}}+1 \end{array}$$ (8)

where $d_{i}^{river}$ is the distance of location *i* to the nearest river, and *l*_*river*_ is an exponential decay of the impact of rivers.

#### Population centres

Closely related to the issue of the roads is the vicinity of major and minor population centres, which provide a market for products and increase the risk of deforestation. Many observational studies simply use the distance to the nearest town or population centre to quantify deforestation risk [63], but this is likely to oversimplify matters, as it neglects the size and population density of the town in question, and moreover does not allow the vicinity of multiple population centres to be accounted for. FOREST-SAGE, therefore, modifies this approach by first calculating a distance weighted population density for each location:

$$\begin{array}{c} {\bar{p}}_{i}=\frac{\sum_{j=1}^{N_{\Omega }}p_{j}e^{-d_{i,j}/l_{p}}}{\sum_{j=1}^{N_{\Omega }}e^{-d_{i,j}/l_{p}}} \end{array}$$ (9)

where *p*_*j*_ is the population density in the grid-cell *j* and *l*_*p*_ is the e-folding distance weighting. The risk factor associated with a grid-cell location due to weighted population density is then represented as:

$$\begin{array}{c} r_{i}^{pop}=\left(k^{pop}-1\right)\left(1-e^{-\bar{p_{i}}/d_{p}}\right)+1 \end{array}$$ (10)

where *d*_*p*_ is the e-folding increase with population density.

The distance weighted population factor allows the distance and market size/land value to be generically incorporated, since land value is related directly to both factors. A location close to a small urban centre will have a similar deforestation risk as a location at a larger distance from densely inhabited metropolis. Nevertheless, deforestation and land-use change in the vicinity of urban centres can have a wide range of causes, the diversity of which is obviously neglected in this approach.

#### Protected areas and logging concessions

Protected areas, national parks and logging concessions are simply treated. The risk factor $r_{i}^{park}$ is set to a constant value equal to *k* ^*park*^ if point *i* is found within a national park. This factor represents the ratio of the decrease in deforestation risk associated with the respective designation status, and thus $r_{i}^{park}$ is set to unity if location *i* is not located in a park. Logging concessions are treated similarly, with $r_{i}^{log}$ set to *k* ^*log*^, representing the increase in deforestation risk associated with logging concessions. For example, if designation of an area as a logging concession inside a certain macro region is thought to make deforestation four times more likely then *k* ^*log*^ is set to 4.0 for all points inside concessions. The value for *k* ^*park*^ on the other hand should take a value smaller than 1.0 if policy is effectively enforced. Policy enforcement and policing of illegal logging activities varies greatly from country to country, and even potentially on a sub-national scale. Likewise, conflict and natural disasters can change policy enforcement drastically as a function of time. Concerning this spatial variation, if a regional user has explicit knowledge of policy enforcement in a particular country or district, this can be incorporated by defining a separate macro-region in FOREST-SAGE and adjusting these risk factors appropriately for this region.

#### Reforestation

Reforestation can significantly offset deforestation rates [63]. Reforestation patterns are often spatially distinct from deforestation [33], with the degree and rate of reforestation strongly depending on the cause of the clearance [64]. Representing this process is not straightforward as it depends both on the potential vegetation (i.e. the vegetation that would exist on a given area for given climatic conditions in the absence of major disturbances) and also on the motivation for deforestation. For example, clearance for permanent agriculture, ranching or urban expansion may lead to limited or no regrowth, whereas shifting non-permanent agriculture can lead to regrowth after a period of years, and clearance for wood harvesting is followed by immediate regrowth [64]. The regrowth algorithm converts the PFT to the categories that are associated with natural forest cover in the location in question using a potential vegetation coverage dataset as the reference that can be derived from a model source (CLM) or by observations (MODIS). In the present demonstration, the potential vegetation cover $f{}_{t}$ is given by the maximum value of forest cover of the MODIS retrievals in the Congo region at 86%. The natural forest cover is then $f{}_{t}$ reduced in each location *i*, to account for the possibility of permanent LUC without regrowth. For this, the assumption is made that permanent deforestation is more likely to occur in the locations with higher weighted population densities, such that the modified potential forest cover is given by $f_{t}e^{-\bar{p_{i}}/\tau_{rp}}$, where *τ*_*rp*_ determines how the likelihood of permanent deforestation changes as a function of weighted population density. Thus the algorithm specifies the regrowth rate as:

$$\begin{array}{c} A_{{}_{i}}=\frac{f{}_{t}e^{-\bar{p_{i}}/\tau_{rp}}-f{}_{i}}{\tau_{r}} \end{array}$$ (11)

where *A*_*i*_ is set to zero if *f*_*i*_ exceeds the modified potential forest cover and *τ*_*r*_ specifies the regrowth timescale. One difficulty in discerning regrowth from remote sensing observations is that it is often difficult to distinguish forest regeneration from deforestation and degradation if the two are co-located [65], as occurring with regeneration of secondary forest in regions of rotational farming.

#### Stochasticity

In order to sample uncertainty in the model settings, the FOREST-SAGE model allows the application of perturbed physics ensembles (PPEs), generated by varying the single values of parameters over time in FOREST-SAGE ensemble member during simulations. By perturbing FOREST-SAGE model physical parameters within plausible ranges (±10%) to create different variants, it is possible both to sample model uncertainty and to evaluate model performance with observations. In addition, in order to increase the ensemble spread of FOREST-SAGE to take into consideration the spatial uncertainties, a stochastic perturbation to model physics has been added to the percentage tree cover matrix using a pseudo-random number generator [66].

### 2.2 Experiment setup

In order to evaluate the FOREST-SAGE model and test its parameter sensitivity, a simple idealized experiment in Central Africa has been conducted. The experiment uses satellite data from MODIS to initialize the model in 2001, and then integrates the model forward in time for one decade to evaluate whether and how well the model can reproduce the broad spatial patterns of deforestation. The only parameter that is specified from the satellite observations is the mean linear deforestation rate over the region; no spatial information is used and instead all of the parameter settings relating to spatial drivers are taken directly from literature where available.

#### Congo region

The region simulated lies between 3.5S to 3.5N in latitude and 18.75E to 25.5E in longitude (Fig 3). This area corresponds to the core of Congo Basin, characterized by the presence of evergreen tropical forest [67] with swamp forest present along the rivers. The forest typically is very dense and often precludes the development of shrubs and grasses [67].

The Congo presently is subject to a low deforestation rate relative to other tropical forests [68], a mere 0.01% per year presently according to the MODIS data used in this study. However, the Congo is the only rainforest where annual deforestation rates are rising [33]. Moreover, in the Central Africa an increase of LUC can be expected in the following years due to both the high rate of population growth, ranging from 2.5–3.5% *year* ^−1^, [33] and to a network of more than 50.000 kilometers of new logging roads [25]. The Congo Basin has a forest area of around 2 million square kilometers representing about 18% of the world’s tropical forests [69] and forest sector activities contribute to 3–8% of the gross domestic product (GDP) in Central Africa [70].

#### FOREST-SAGE input data

The model uses freely available GIS datasets for the local drivers of roads and rivers [71], population density [72], protected areas and logging concessions [25], with futher details provided in Table 1. Of particular interest for the Congo region are the data concerning parks and logging concessions. Despite a lower deforestation rate compared to other tropical forests, the Congo rainforest ecosystem is likely to become increasingly fragile as result of enhancing commercial logging. The data for protected area (PAs) was derived from the World database on Protected Areas (WDPA, version release 2010) that contains both spatial and attribute data with the shapefiles projected to the experiment resolution of 5 km. Although the level of designation of park is also available in the database (local, national or international) and FOREST-SAGE allows the user to specify a different value of *r* ^*park*^ for each level, no information on the protection level as a function of park status was found, and *r* ^*park*^ is constant for all parks. Currently, more than 600,000 km^2^, 30% of forest are under logging concessions [25]. Logging concessions have a positive feedback on the deforestation rate because logging companies build roads through the rainforest and clear large areas of forest increasing accessibility to remote areas. The logging concession dataset was provided by the Woods Hole Research Center [25].

**Table 1:** Details of the input data used to initialize the model.

**Input**	**Reference**	**Source**	**Brief Description**
Forest Cover	[75]	http://glcf.umd.edu/data/vcf/	The dataset is derived from all seven bands of the MODerate-resolution Imaging Spectroradiometer (MODIS) sensor onboard NASA’s Terra satellite.
Roads and Rivers	[71]	http://www.diva-gis.org/gData	The database is derived from the Digital Chart of the World (1:1000000 scale) and it is divided into 2094 tiles that represent 5-degree by 5-degree area of the globe.
Population	[72]	http://www.afripop.org/	AfriPop dataset provides per-grid square estimates of numbers of people at 1 km of horizontal resolution. The population data primarily comes both from censuses 1993–2010 and from settlements maps derived from Landsat imagery.
Parks	IUCN and UNEP. The World Database on Protected Areas (WDPA). UNEP-WCMC.	http://www.protectedplanet.net http://www.wdpa.org/	The World database on Protected Areas (WDPA) provides data for protected areas (PAs) by ArcGIS shapefiles in polygon format which are converted to a binary map at the resolution of FOREST-SAGE maps. The release date of the version incorporated is of 2010.
Loggings	[25]	http://www.whrc.org/	The logging concession dataset was provided by the Woods Hole Research Center

### 2.3 Strength and scale of LUC drivers

In as far as is possible, the constants that define relative importance and spatial impact of each driver have been taken from studies in the literature (Table 2), which are not always available in the focus region of Central Africa. For many of these factors our literature review only revealed a single or a limited number of studies, or in some cases such as rivers, none at all, and thus the uncertainty in their specification is considerable.

**Table 2:** Summary of the 729 ensemble experiments set by the choice of different valued parameters. The three experiments pointed out have been chosen using as metric the best spatial correlation (experiment 1), the minimum BIAS (experiment 2) and the minimum RMSE (experiment 3).

Driver	Reference Articles	Ensemble Mean	Exp. 1(S2 File)	Exp. 2(S3 File)	Exp. 3(S4 File)
Roads	[17, 37, 59, 60]	𝒪 10-45-100 km	𝒪 100 km	𝒪 10 km	𝒪 100 km
Population	[9, 16, 18, 33]	8-30-100 people per km^2^	100 people per km^2^	8 people per km^2^	100 people per km^2^
Rivers	[61, 62]	𝒪 1-5-10 km	𝒪 1 km	𝒪 5 km	𝒪 1 km
Parks	[33, 62]	0.5-0.8-1.0 (50-75-100%)	No Impact (1.0)	No Impact (1.0)	No Impact (1.0)
Logging Concessions	[25, 33]	1.0-1.3-2.0 (100-130-200%)	130% (1.3)	200% (2.0)	130% (1.3)
Reforestation	[64, 81, 83]	30-40-60 years^−1^	60 years^−1^	60 years^−1^	40 years^−1^
R		0.61	0.68	0.56	0.68
RMSE		0.26	0.24	0.26	0.23
BIAS		-0.009	0.005	0.0001	-0.007

To investigate the impact the parameter setting uncertainty may have, the FOREST-SAGE parameter settings are explored in a simple, two-stage multiple parameters ensemble simulation setup. In the first stage, an ensemble of 729 experiments is conducted, given by the combination of the six model parameters deemed to have the larger uncertainty, each of which is allocated one of three possible values in each experiment. The parameter values are chosen to sample the range given in the literature, or an estimated range of uncertainty where only a single observed value is available. Details are specified in Table 2. The best three experiments in terms of spatial correlation, minimum bias and minimum RMSE are also given in the table (data are available in Supporting Information: S2 File, S3 File and S4 File, respectively). The focus is on six key parameters in this first step due to the prohibitive computational cost of investigating all possible parameter values of the full FOREST-SAGE parameter-set in a multiple parameters ensemble framework.

**Fig 5:**
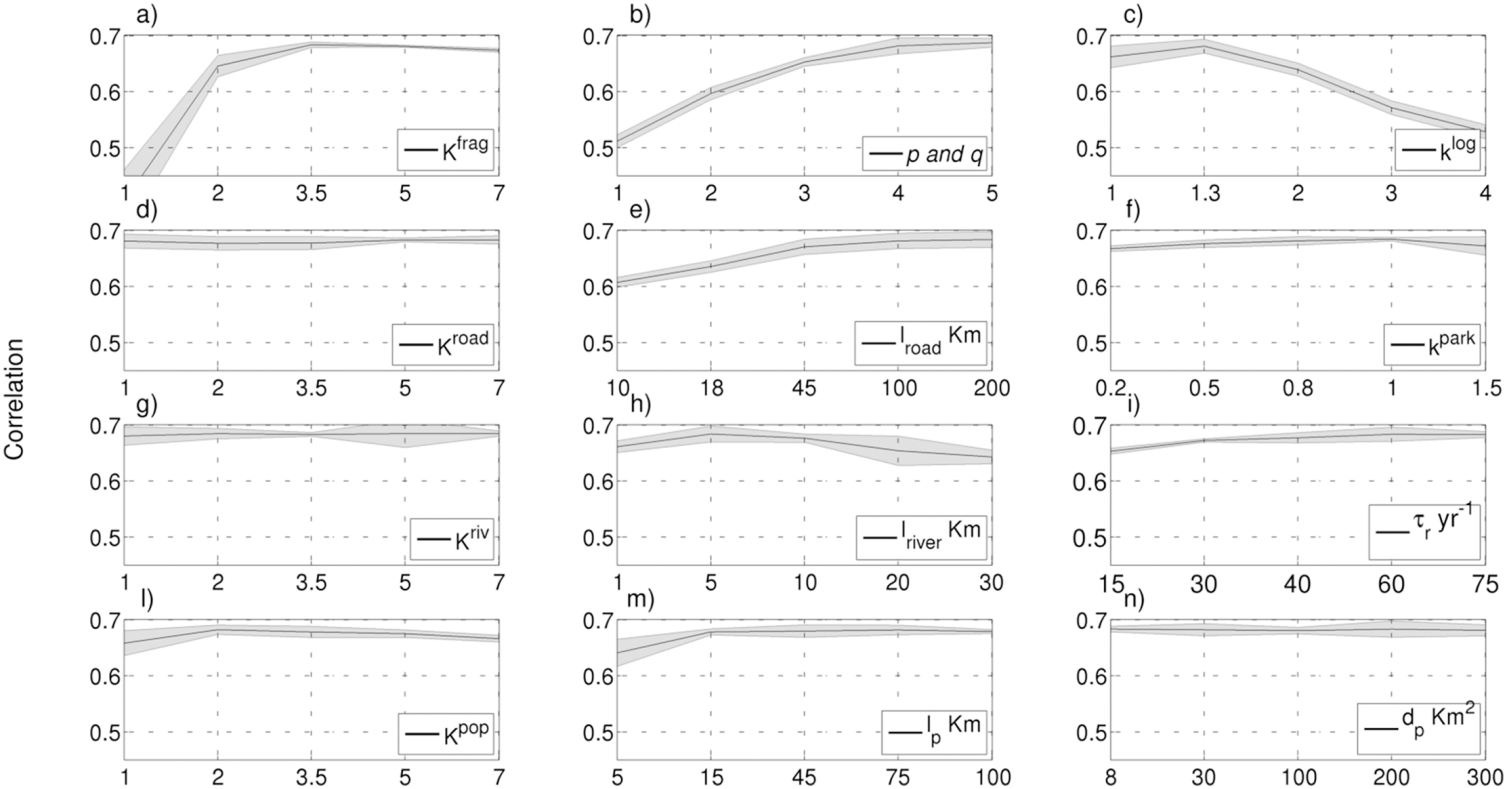
FOREST-SAGE model sensitivity. The 240 sensitivity experiments are performed changing each time a single valued parameter setting the experiment 1 as reference and comparing the results (averaged at 50 km) against the MODIS trend 2001-2010. To evaluate the model sensitivity a perturbed physics ensembles (PPEs) have been conducted and, also, in order to increase the ensemble spread of FOREST-SAGE and to take into consideration the spatial uncertainties, a stochastic perturbation to model physics has been added. On the *y*-axis the spatial correlation is shown, while on the *x*-axis the parameter values are given. The panels *a*−*c*−*d*−*f*−*g*−*l* are referred to the weights of fragmentation, logging, road, park, river, population respectively. The panel *b* is referred to the *p*−*q* beta distribution parameters, while the panel *i* is referred to the reforestation rate. Moreover the panels *e*−*h*−*m* are referred to the e-folding spatial scale of roads, rivers, population while the panel *n* is referred to the e folding increase with population density.

Once the best set of parameter settings are achieved for these six variables, the second stage involves further sensitivity experiments (Fig 5) in which the full FOREST-SAGE parameter set are around this initial best setting. Starting from the constant values used in the experiment 1 (Table 3), chosen as benchmark due to greatest spatial correlation between modelled and observed forest cover change, a single value parameter has been modified in every run: the results, averaged at 50 km, are then compared with MODIS-VCF, adopting as metrics the change in spatial correlation, mean bias or root mean square error. Each of the twelve FOREST-SAGE parameters is set to one of five values. In order to increase the ensemble spread, simulations with both PPEs and a stochastic perturbation to model physics have been included (Fig 5).

**Table 3:** Summary of the constant values used in the experiment 1 of Table 2. The experiment 1 outputs have been attached in Supporting Information (S2 File).

**Variable**	**Description**	**Value**
*k* ^*roads*^	Weight of the Roads	3.5
*k* ^*pop*^	Weight of the population	3.5
*k* ^*rivers*^	Weight of the Rivers	3.5
*k* ^*frag*^	Weight of the fragmentation	3.5
*k* ^*log*^	Weight of the logging area	1.3
*k* ^*park*^	Weight of the protected area	1.0
*p*—*q*	Beta distribution parameters	4-4
$l_{road}$	e folding spatial scale roads	100 km
$l_{river}$	e folding spatial scale rivers	1 Km
*l*_*p*_	e folding spatial scale population	15 Km
*d*_*p*_	e folding increase with population	100 people km ${}^{-2}$
*τ*_*rp*_	Population sensitivity of permanent deforestation	200 people km ${}^{-2}$
*τ*_*r*_	Reforestation rate	60*yr* ^−1^

Concerning the values used in the two-stage, multiple parameters ensemble framework, from the complete FOREST-SAGE parameter set, the impact of roads is perhaps one of the best documented effects in the literature, but despite this, the uncertainty is high. The spatial impact of roads, for example, can range from 𝒪 < 10 km [59] along newly built roads, up to 𝒪 100 km [60]. Instead smaller distances has been chosen for the rivers (ranging from 1 to 10 km).

Setting the risk factors for logging concession/national parks is challenging, as the resulting LUC it depends on the time-frame over which the logging concession resources will be extracted, and the level of policy enforcement for protected areas. Thus it is important to evaluate the sensitivity of FOREST-SAGE to different risk settings. The allocation of protected status can reduce deforestation rates by a factor of 50% in this region [33], whereas the occurrence of logging concessions does not have a clear impact on deforestation rates [33]. For the logging concessions a set of values ranging from “no impact”, to “high impact” (from 1.0 to 4.0) has been chosen and similarly for the protected area different levels of policy enforcements have been tested.

Regarding the population drivers, starting from Ernst et al. [33], several tests have been performed, assuming a critical population density value ranging from 8 to 100 people per km^2^, while for the population spatial impact *l*_*p*_ values ranging from 5 to 30 km have been selected. For the reforestation time *τ*_*r*_ values between 15 years and 75 years have been chosen and, in absence of a high resolution potential vegetation map over the Congo area, a value equal to the maximum forest cover value (i.e *f*_*i*_=86%) has been chosen as *f*_*t*_. The *p* and *q* parameters have been changed in order to modify the shape of the beta function ranging from the assumption of an equal probability deforestation at different percentage of forest cover (*p* and *q* equal to 1.0) to a high risk value centered around the 50% of forest cover (*p* and *q* equal to 5.0).

While it is relatively easy to find parameter values for the spatial distribution of the deforestation risk (Table 2), instead the sources of information for the *k* parameters are scarce and show a wide range of values [6, 16, 59]. In addition the deforestation risk calculated from remote sensing is often overestimated due to the complexity of discrimination between the individual forcing acting on a single pixel (i.e the risk is often the sum of different local drivers). Nevertheless, in order to verify the relative importance of a driver compared to the others a wide range of possible values has been adopted, assuming values between 1.0 and 7.0.

### 2.4 Satellite data

While FOREST-SAGE is designed to be interfaced directly with land-surface models such as CLM, for this evaluation the model has been initialized with land cover information derived from MODerate-resolution Imaging Spectroradiometer Vegetation Continuous Field (MODIS-VCF) [73, 74]. Specifically, the percentage tree cover dataset (collection 5 version 1 release data, MOD44B) [75] has been used as input. The MODIS-VCF product combines MODIS and LandSat information to produce an annual dataset (presently available from 2000 to 2010) with 250 meters spatial resolution of the following parameters: Percent tree cover, Quality Assurance (QA) bad data flag, QA cloudy data and standard deviation of models. For full details of the retrieval algorithm see Hansen et al. [76, 77].

The percent tree cover describes the percent canopy cover in each pixel (values ranging from 0 to 100) and is accompanied by the QA bad data that is a quality flag defining the pixels having poor/good quality because of cloud coverage, high aerosol, cloud shadow or view zenith > 45°, while the QA cloudy data is a quality flag focused on the cloud coverage only. The Collection 5 VCF products are still under active development and the treatment of cloud-affected pixel in the retrieval algorithm is a remaining challenge (Dimiceli C. M., personal communication) and is a caveat of this work. For each year, pixels with a bad-quality flag exceeding 12.5% are rejected from the analysis.

The Congo Basin, in common with other tropical areas, is characterized by high cloud coverage [8], especially near the west coast, which is the main source of uncertainty for the forest evaluation from satellite [78]. In 2000 the area affected by low quality was considerably greater than for the subsequent years (almost half of the Congo Basin area was not available according to the QA criterion applied) and thus FOREST-SAGE was initialized in the year 2001.

The MODIS product is presently available until 2010, and thus the FOREST-SAGE model is integrated from 2001 to 2010, with the domain mean deforestation rate of 0.01% applied to match the mean observed rate using the MODIS-VCF data. It is acknowledged that 9 years is a relatively short period for examining deforestation trends, and thus with this dataset it is presently only possible to calculate the annual linear trend over the decade. In any case, year-to-year variability in the leaf canopy due to climate variations, along with changing spatial availability of satellite data due to changing cloud cover precludes the examination of year-by-year variations in land cover.

The MODIS data is aggregated to 5 km resolution, which is the resolution used for the model integrations. This resolution is on the borderline of resolving the impact of deforestation drivers such as vicinity to roads and cities which have been assessed to have an e-folding impact on the order of 10 km [36, 37]. However, as deforestation is essentially a stochastic process (i.e roads increase deforestation, but not uniformly along their length, but rather in sporadic settlements), the model outputs have been averaged to 50 km for the evaluation statistics. The linear regression fit to the simulated land cover change is then compared to the linear regression fit to the satellite observed LUC in each pixel that passes the data quality criterion.

### 2.5 HYDE benchmark model

In order to gauge the performance of the FOREST-SAGE model over the Congo region, it is compared to benchmark LUC scenarios provided by the HYDE model database [52]. The HYDE database consists of two components. The first is a historical database of LU information including proxies such as population density and distribution. A set of statistical rules are used in the model to convert this information to an annual, gridded map of land use for the period 1500–2005 [79].

The second component of the database was the use of the model to convert the future LUC scenarios to a consistent gridded format. These were provided by the four IAMs: MESSAGE, AIM, GCAM and IMAGE that are respectively expressing the Representative Concentration Pathways (RCPs) defined by their total radiative forcing: RCP8.5 [49], RCP6.0 [48], RCP4.5 [47] and RCP2.6 [46]. Hurtt et al. [52] describes the process of how the historical and scenario periods are merged to provide a smooth, harmonized set of LUC scenarios for 1500 to 2100.

The HYDE land-use outputs do not provide direct forest cover, but instead are expressed in terms of primary and secondary land cover proportions, in addition to urban, pasture and crop coverage. Thus, the forest fraction is derived by combining the primary and secondary fraction with a map for potential vegetation coverage [80]. The forested area has been calculated as the sum of primary and secondary forest.

The MODIS observational period of 2001 to 2010 spans the historical and future scenario datasets. Therefore four HYDE-derived estimates of the 2001–2010 change in forest cover are derived, which are identical for the historical period 2001–2005, and differ substantially for the period 2006–2010 according to the scenario used. The change in land cover for the historical period 2001–2005 is effectively zero for the region in question, and the 2001–2010 trends are almost entirely due to the scenario employed.

## 3 Results

### 3.1 Parameter sensitivity for the Congo simulations

Here, the sensitivity of the FOREST-SAGE simulations to the model parameter settings in the two-stage multiple parameters ensemble simulations is reported.

The multiple parameters ensemble demonstrates that the model is most sensitive to the fragmentation and population *k* values (Fig 5a–5l), it is relatively most insensitive to the roads and river *k* values (Fig 5d–5g). A possible explanation of the weak dependency to the roads/rivers can be associated both to the ubiquity of these networks (Fig 3) and, in the road case, to the large *l*_*road*_ value adopted (Table 3) that decreases the sensitivity to *k* (Fig 4).

The spatial impact of the local drivers plays an important role in the correct detection of the patterns, giving higher correlation when the roads can influence the deforestation up to 100 km, in accordance with Pfaff et al. [60], while the river impact is confined to the first 10 km (after this threshold the correlation starts to decrease). The population spatial impact is almost flat for values higher than 10 km, while, for evaluating the forest cover change the presence/absence of population appears more important than the population density itself. FOREST-SAGE shows the highest sensitivity to the fragmentation, both to the strength of the driver (*k* ^*frag*^) and to the shape of the beta function (Fig 5). A maximum probability deforestation risk centered at 50% of forest cover [57] results in the highest correlation, whereas removing the fragmentation effect altogether (*p* and *q* equal to 1.0) results in the lowest correlation.

**Fig 6:**
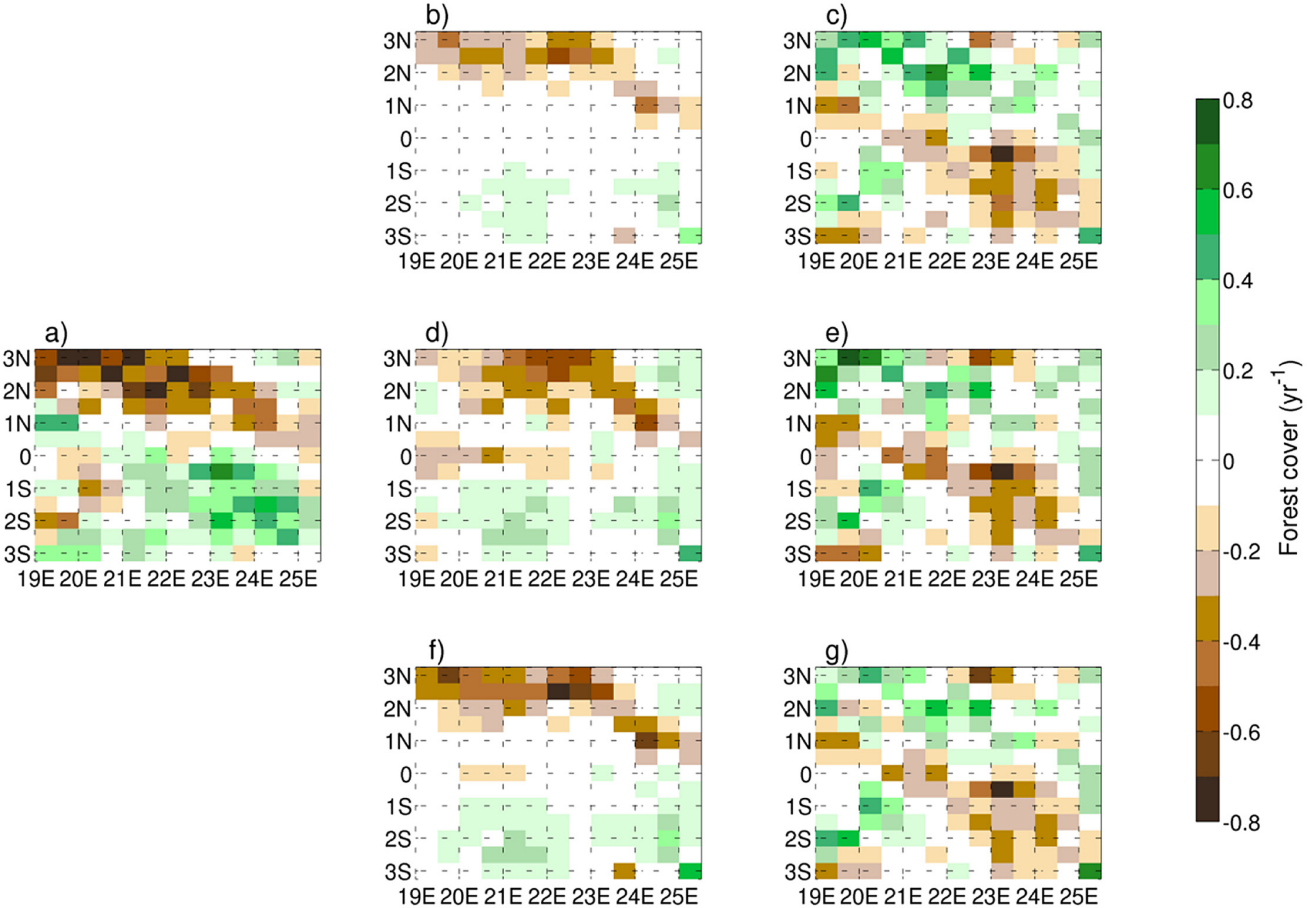
MODIS (panel *a*) and FOREST-SAGE (panels *b*−*d*−*f*) trend years 2001–2010. The displayed panels *b*−*d*−*f* are referred to the FOREST-SAGE experiments 1–3 (Table 2) respectively, while the panels (*c*−*e*−*g*) represent the difference between FOREST-SAGE and MODIS. Negative values indicate a deforestation trend, while positive values an increase in forest cover.

Although the reforestation timescale shows the highest correlation when adopting a time scale of 60 years that is slightly higher than values found in literature [81], setting a time scale of 40 years significantly improves the model’s spatial variance when compared with MODIS observations (Fig 6f). The logging concessions clearly increase the deforestation risk, reaching the highest correlation assuming a deforestation risk of 30% higher for the points inside a logging concession when compared to the surrounding. In contrast, protected areas have a limited impact on the correlation due to their small extent relative to the logging concessions, nevertheless, the analysis suggests that the protection’s impact is less in this area than stated in Ernst et al. [33]. Andam et al. [82] noted that the remoteness of a protected area was often key to its level of protection, rather than policy enforcement, indicating that the risk factor for parks is likely to be rather heterogeneous. Hereinafter the experiment 1 (“S2 File” is available in Supplementary Information) has been designated as the reference for the following comparisons.

### 3.2 Deforestation simulations

**Fig 7:**
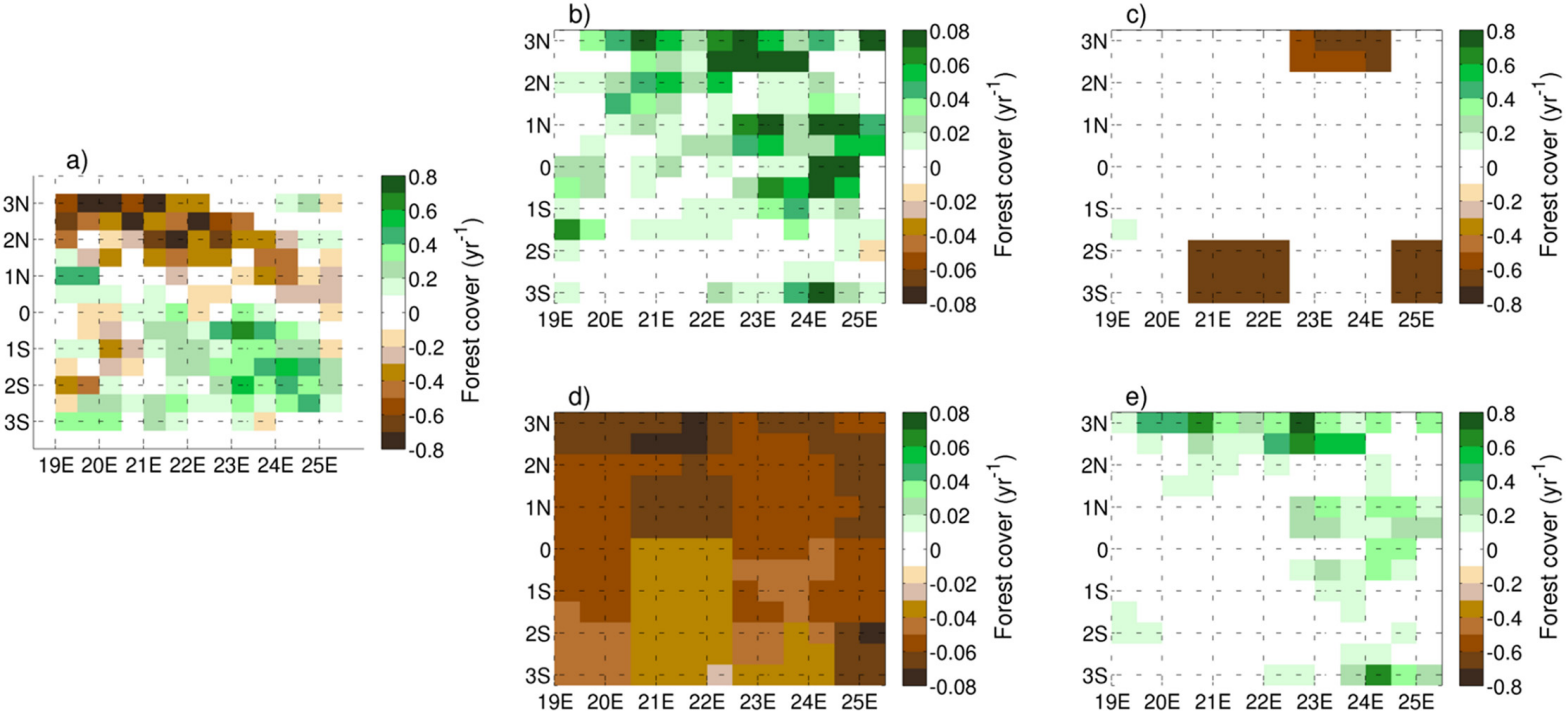
MODIS (panel *a*) and HYDE (panels *b*−*c*−*d*−*e*) trend for years 2001–2010. The displayed panels *b*−*c*−*d*−*e* are referred to the AIM (RCP6.0), IMAGE (RCP2.6), MESSAGE (RCP8.5) and GCAM (RCP4.5) models respectively. Due to the weak magnitude signal of AIM and MESSAGE, panels *b*−*d* are shown with differing color scales.

The spatial patterns of deforestation observed by MODIS and simulated by FOREST-SAGE and HYDE are shown in Figs 6 and 7. There is a good agreement between the FOREST-SAGE output and the satellite retrieval with a maximum spatial correlation of 0.68 (Table 2). The spatial variability appears to be primarily driven by population density and forest accessibility, in particular the deforestation is clearly more accentuated along the Congo river, in accordance with Duveiller et al. [7], and generally in the northern part of the domain where a higher population density is present. Furthermore deforestation appears more active in presence of logging concessions and where the forest cover is not so dense.

In the southern part of the region, where the reforestation process is relatively dominant, the correlation between FOREST-SAGE and MODIS-VCF is lower (R ∼ 0.61) compared to the northern region (R ∼ 0.74). The problem with reforestation process modelling is due both to its complexity [83] and to the lack of a clear link to other forest cover changes [33]. In general the model tends to well reproduce the forest cover trend along the Salonga National Park, slightly overestimating a net increase in forest cover change process along the Salonga National Park (South) and underestimating it along the northern boarder of the same protected area.

Along the Congo River, where the density of population/logging concessions is higher, the model is underestimating the net forest cover change (Fig 6c, 6e, and 6g). Nevertheless, in this first validation attempt, FOREST-SAGE showed the ability to capture the main spatial patterns of deforestation, even if with a slight underestimation of the signal strength.

While the FOREST-SAGE model has obvious deficiencies in the spatial patterns of LUC and their magnitude, the advantage of using local scale drivers for present-day and near-term LUC simulation is apparent if the model is compared to the benchmark simulations of the HYDE model database (Fig 7). Comparing the four harmonized scenarios of LU, none are able to capture the spatial variability or magnitude of the forest cover trend. In addition to the spatial misrepresentation, the forest change also shows a problem in the magnitude of the signal in two scenarios of RCP6.0 and RCP8.5.

Regarding the historical period, it is important to reemphasize the remarks of [79], that while observations and proxy are used as input to the process, the HYDE database is a model product, and does not represent direct observations of land use. It is also recalled that the HYDE LUC for the period 2001–2005 is very limited, and the differences in these projections is entirely due to scenario used. Akkermans et al. [84] called for an improved representation of the RCP deforestation scenarios at least at regional level. This comparison confirms that such improvements can potentially be made in the near-term projections of LUC by employing a model such as FOREST-SAGE at the interface between an broad regional LUC scenario (for example provided by integrating directly the IAM or harmonized HYDE output over a coarse scale), and the climate model, accounting for local-scale drivers. As illustrated in the introduction, this would also have the advantage of being able to integrate the local scale, anthropogenic LUC model on-line with a climate model coupled to a dynamical vegetation model, ensuring that both anthropogenic and natural vegetation changes occur in tandem and in a self-consistent way.

## 4 Discussion

A new model has been introduced, FOREST-SAGE, which is designed to allow anthropogenic land-use change to be fully integrated with the dynamic modelling of vegetation in earth system models, with the ultimate goal of improving the understanding of the role of LUC on the Climate System. The model takes generalized regional scenarios of LUC and disaggregates them to a fine spatial scale accounting for local LUC risk factors (roads, population density, forest fragmentation, logging concessions and national parks) and producing annual changes in plant function types as used by dynamical vegetation models. The risk factors are presently specified for the conversion of primary forest to agriculture/pastoral use, but the aim is to generalize the model to other land use conversions later.

In this first experiment, FOREST-SAGE has been tested in an ‘offline’ mode, in an attempt to simulate recent trends in forest cover in Central Africa, using a recently developed forest cover retrieval MODIS-VCF to initialize the model in 2001, and then to evaluate the simulations for subsequent years. All the local risk factors were obtained from the open literature and no information is used from MODIS-VCF in the model, with the exception of its initialization. As many of these factors are highly uncertain, multiple experiments were conducted in a grand-ensemble. This initial experiment demonstrated a broad ability of the model to reproduce spatial patterns of deforestation and forest recovery, and the model was particularly sensitive to parameters regarding the population, fragmentation and logging concessions, while the ubiquitous transport network had a lesser effect indicating the need to improve the model to differentiate between minor and major routes, and also to account for the presently neglected topography and soil quality, which may determine where along major routes settlements are most likely to occur. Further refinements that could benefit the model include the identification of active logging concessions in the area and taking into account the effectiveness of the parks in reducing deforestation. The most significant limitation of the model for its use in climate projections is the use of static maps of risk factors, rather than attempting to project the impact of future population growth on the transport network [29] and population centres. This shortcoming is in common with many present LUC models.

FOREST-SAGE is not necessarily new in its treatment of the local deforestation risk factors [43] but attempts to provide a model to interface knowledge of local scale drivers of LUC in a generalized framework that can be interfaced directly with dynamic vegetation models such as the commonly used land-surface climate model. While the generality of the model required for climate modelling implies that the model may not necessarily match the ability of a regionally-derived regression model for past LUC, the flexibility of the approach implies that it can easily be improved by tuning the relationships over local or regional scales, and (the approach) extended to include other drivers and factors presently neglected that may be regionally important. The current state-of-the-art in harmonized land-use scenarios is represented by the History Database of the Global Environment (HYDE) dataset [52] that provides historical and future land-use data with future projections ESMs. However, each modelling system must then translate these projections into their respective PFTs, and the use of offline projections implies that they are not always consistent with the dynamic vegetation model’s local vegetation cover, and each modelling centre must introduce their own set of rules to resolve such conflicts [50, 51]. By using FOREST-SAGE to translate global anthropogenic land-use scenarios to earth system model grid-scale land cover on-line in a fully coupled way, such feedbacks can be incorporated and the self-consistent treatment of LUC and vegetation is facilitated. A future manuscript will demonstrate the model being used to integrate anthropogenic LUC projections from the HYDE model seamlessly to a coupled climate-land surface modelling framework using an active DGVM.

## Supporting Information

S1 File FOREST-SAGE source code. FOREST-SAGE version 1.0 source code combined with FortranGIS library package containing shapelib module. The model is written in Fortran90 and requires Fortran and NetCDF libraries installed.
(ZIP)

S2 File FOREST-SAGE best correlation experiment. The file has been initialized by MODIS-VCF 2001 using the setting of experiment 1 and contains 10 years of FOREST-SAGE simulations at 5 km of horizontal resolution.
(NC) Click here for additional data file.

S3 File FOREST-SAGE minimum BIAS experiment. The file has been initialized by MODIS-VCF 2001 using the setting of experiment 2 and contains 10 years of FOREST-SAGE simulations at 5 km of horizontal resolution.
(NC) Click here for additional data file.

S4 File FOREST-SAGE minimum RMSE experiment. The file has been initialized by MODIS-VCF 2001 using the setting of experiment 3 and contains 10 years of FOREST-SAGE simulations at 5 km of horizontal resolution.
(NC) Click here for additional data file.
